# BRD2 bromodomain-mediated regulation of cell state plasticity modulates therapy response in glioblastoma

**DOI:** 10.1093/neuonc/noaf169

**Published:** 2025-07-19

**Authors:** Raghavendra Vadla, Brett Taylor, Yohei Miyake, Benjamin Lin, Daisuke Kawauchi, Shunichiro Miki, Nidhi Nathwani, Brandon M Jones, Yashpreet Kaur, Abhinaba Banerjee, Philip Pham, Jonathan Tsang, Albert Baldwin, David A Nathanson, Donald P Pizzo, C Ryan Miller, Frank B Furnari

**Affiliations:** Division of Regenerative Medicine, Department of Medicine, University of California San Diego, La Jolla, California, USA; Medical Scientist Training Program, University of California, San Diego, La Jolla, California, USA; Division of Regenerative Medicine, Department of Medicine, University of California San Diego, La Jolla, California, USA; Division of Regenerative Medicine, Department of Medicine, University of California San Diego, La Jolla, California, USA; Medical Scientist Training Program, University of Alabama at Birmingham, Birmingham, Alabama, USA; Department of Pathology, Division of Neuropathology, Heersink School of Medicine, University of Alabama at Birmingham, Birmingham, Alabama, USA; Division of Regenerative Medicine, Department of Medicine, University of California San Diego, La Jolla, California, USA; Division of Regenerative Medicine, Department of Medicine, University of California San Diego, La Jolla, California, USA; Division of Regenerative Medicine, Department of Medicine, University of California San Diego, La Jolla, California, USA; Division of Regenerative Medicine, Department of Medicine, University of California San Diego, La Jolla, California, USA; Division of Regenerative Medicine, Department of Medicine, University of California San Diego, La Jolla, California, USA; Department of Bioengineering, University of California, San Diego, La Jolla, California, USA; Division of Regenerative Medicine, Department of Medicine, University of California San Diego, La Jolla, California, USA; Departments of Molecular and Medical Pharmacology, University of California, Los Angeles, California, USA; UNC Lineberger Comprehensive Cancer Center, University of North Carolina School of Medicine, Chapel Hill, North Carolina, USA; Department of Bioengineering, University of California, San Diego, La Jolla, California, USA; Department of Pathology, University of California San Diego, La Jolla, California, USA; Department of Pathology, Division of Neuropathology, Heersink School of Medicine, University of Alabama at Birmingham, Birmingham, Alabama, USA; Division of Regenerative Medicine, Department of Medicine, University of California San Diego, La Jolla, California, USA

**Keywords:** BET inhibitors, BRD2, glioblastoma, mesenchymal, NF-κB signaling

## Abstract

**Background:**

Glioblastoma (GBM) displays remarkable cell state plasticity, a major contributor to therapeutic resistance and tumor progression. While epigenetic mechanisms play a central role in driving this plasticity, the key regulators remain poorly understood, and developing effective therapeutic strategies targeting them has been challenging.

**Methods:**

We investigated the role of BRD2, a key regulator of NF-κB-mediated mesenchymal (MES) transition, using GBM patient-derived xenograft cell lines, CRISPR-mediated knock-in/knockout approaches, RNA-seq, and in vitro and in vivo modeling. BET inhibitors were employed to target MES gene expression and sensitize GBM to radiation therapy.

**Results:**

We found that PTEN loss induces RelA chromatin localization and acetylation-mediated recruitment of BRD2 to the MES gene promoters. BRD2 binding is essential for maintaining MES gene expression and phenotype. Genetic ablation or loss-of-function mutation of BRD2 bromodomains reverses MES transition, enhances radiation sensitivity, and improves survival in orthotopic xenograft models. Additionally, treatment with a brain-penetrant BD2-selective inhibitor suppresses the MES phenotype and increases radiation sensitivity of GBM stem cells in vitro.

**Conclusion:**

Our study identifies BRD2 as a key mediator of MES transition in GBM, with its bromodomains playing a crucial role in driving cell state plasticity. Targeting BRD2 with BD2-selective inhibitors offers a promising therapeutic strategy to overcome radiation resistance and improve outcomes for GBM patients.

**Key points**

- BRD2 regulates NF-κB-mediated mesenchymal transition in GBM.
- BET-BD2 inhibitors inhibit MES transition and sensitize GBM to radiation.

**Importance of the Study**

Mesenchymal transition in GBM is strongly linked to therapy resistance and disease recurrence. While NF-κB drives MES gene expression, direct targeting remains unfeasible due to the lack of therapeutically tractable targets. Using CRISPR-based NF-κB modulation, we identified BRD2 as a key regulator of the MES transition and uncovered a mechanism in which GBM cell state plasticity depends on BRD2 bromodomains. Our study provides a compelling rationale for combining BD2-specific inhibitors with standard GBM therapies, highlighting a promising strategy to overcome therapy resistance and improve patient survival.

Glioblastoma (GBM) presents a significant challenge in cancer treatment due to its remarkable ability to switch between different cellular states, a phenomenon known as cell state plasticity.^1^ Despite aggressive treatments involving surgical resection, radiotherapy, and chemotherapy, recurrence is almost inevitable.^2,3^ Bulk RNA sequencing (RNA-seq) analyses have identified 3 transcriptomic subtypes in IDHwt GBM: classical (CL), mesenchymal (MES), and proneural (PN), with the MES subtype particularly linked to poorer outcomes and survival rates.^1,4^ The MES subtype is characterized by higher therapy resistance, increased invasion, and the presence of tumor-associated macrophages/microglia (TAMs).^5–7^ Studies indicate that IDHwt tumors with a MES recurrence have a significantly shorter interval between surgeries compared to nonmesenchymal recurrences.^8^ Moreover, tumors tend to transition to the MES subtype upon recurrence.^9^ The biological mechanisms driving this MES transition remain poorly understood.

Multiple oncogenic pathways have been associated with MES transition in GBM.^10^ Among them, activation of the NF-κB pathway is crucial in promoting this transition.^6,11^ NF-κB signaling enhances the expression of key MES genes as well as various cytokines, chemokines, and growth factors, which collectively modulate the tumor microenvironment and contribute to the enhanced MES phenotype.^12^ PTEN is a negative regulator of the NF-κB pathway and is frequently mutated or deleted in GBM.^13^ However, the connection between PTEN and the NF-κB pathway as well as how PTEN may function upstream to influence NF-κB signaling during MES transition in GBM, remains poorly understood.

Bromodomain and Extra Terminal (BET) proteins, which include BRD2, BRD3, BRD4, and BRDT, function as readers of acetylated proteins and play a critical role as coactivators in oncogenic transcriptional programs.^14^ BRD4 binds to acetylated RelA at lysine 310, thereby promoting the transcription of inflammatory genes.^15^ BRD2 and BRD4 are overexpressed in GBM,^16^ but their specific roles in promoting NF-κB-mediated gene expression in GBM remain unclear. BET proteins are involved in epithelial to mesenchymal transition (EMT) in other cancers,^17,18^ but their roles in driving MES transition in GBM remain unexplored. Given the growing interest in developing bromodomain-specific inhibitors that can cross the blood–brain barrier,^19^ understanding the functions of the RelA/BET complexes in GBM is crucial for designing effective therapeutic strategies.

Herein, we uncover the crucial role of BRD2 and its bromodomains as key modulators of the MES transition in GBM downstream of PTEN/NF-κB signaling. We further demonstrate the effectiveness of BRD2-specific inhibitors in blocking this transition, prolonging mouse survival, and enhancing sensitivity of GSCs to ionizing radiation (IR).

## Materials and Methods

### Cell Culture and Reagents

Parental U87MG were obtained and cultured as described previously.^20^ GSCs were obtained as previously described.^21^ GSCs were cultured in DMEM + GlutaMAX medium supplemented with B27 (GIBCO/Life Technologies) and 20 ng/mL human recombinant EGF and 20 ng/mL bFGF. All cells were incubated at 37 °C and 5% CO_2_. GSK620 and Ipatasertib were purchased from MedChemExpress. ABBV-744 was provided by Andrew Shiau (UCSD). pLV-IκB-SR vector and pGL4.32[luc2P/NF-κB-RE/Hygro] vector were obtained as described previously.^22^ Mission shRNA constructs targeting BRD2 were purchased from Sigma.

### Protein Subcellular Fractionation and Western Blotting

Subcellular Protein Fractionation Kit for Cultured Cells (ThermoFisher Scientific) was used to separate different protein compartments within cells. Cytoplasmic, nuclear, and chromatin-bound proteins were extracted according to the manufacturer’s instructions (#78840). For whole cell lysates, cells were lysed in RIPA buffer. The following antibodies were used, anti-BRD2 (Cell Signaling Technologies (CST), #5848), anti-RelA/p65 (CST, #8242), anti-BRD4 (CST, #13440), anti-GAPDH (CST, #2118), anti-TBP (CST, #44059), anti-PTEN (Millipore, #04-035), anti-H3 (Novus Biologicals, #NBP1-61519), anti-AKT (CST, #9272), anti-phospho-AKT Thr308 (CST, #9275), anti-acetyl-NF-κB p65 (CST, #3045), p300 (Santa Cruz, #sc-48343), CD44 (CST, #37259), and ANXA1 (CST, #32934).

### Generation of CRISPR-engineered Cell Lines

pSpCas9(BB)-2A-GFP (px458) plasmid was used to generate endogenous RELA K310R and PTEN KO GSCs as described.^23^ See Supplementary Methods for details.

### Immunofluorescence

GSC11 cells expressing wild-type or mutant RelA were seeded on coverslips and treated with TNF-α (20 ng/mL) for 20 min. Cells were then washed with PBS, fixed in 4% PFA for 15 min at room temperature (RT), and permeabilized with 0.3% Triton X-100 for 10 min at RT. Coverslips were then blocked by incubation in 2% BSA in PBS at RT for 30 min, followed by 5% donor bovine serum (ThermoFisher Scientific) for 20 min. Blocked coverslips were then probed with RelA antibodies diluted in PBS containing 2% BSA and incubated in a humified chamber overnight at 4 °C. Next, fluorochrome-conjugated secondary antibody (Invitrogen, #A32731, working concentration 1:1 000) diluted in PBS containing 2% BSA was added and the coverslips were incubated for 2 h at RT in the dark. Coverslips were mounted with Fluoro-Gel II with DAPI (Electron Microscopy Sciences). Imaging was conducted using a Keyence microscope at 20× or 40× magnification.

### RNA-seq Data Analysis

Paired-end 150 bp RNA-seq was performed by Novogene Corporation Inc, Sacramento, CA. Fastq file quality was ensured with FastQC, and reads were aligned to the hg38 index using STAR. Alignment quality was confirmed by assessing the final STAR logs. Index and bigwig files were generated from the BAM files with samtools and bamCoverage, respectively. Each BAM file was viewed in IGV to assess the quality and features of mapped reads. The Subread package was used to generate count files, and the raw counts were used as input files to define differentially expressed genes (DEG). Genes with less than 50 total counts across all samples were filtered out for DEG analysis. All the above packages were downloaded and maintained with the Conda package manager. In R, the DESeq2 package was used for DEG analysis. Hierarchical clustering was visualized using heatmaps generated by pheatmap, and volcano plots were created with EnhancedVolcano. Initial DEGs were identified using a Benjamini–Hochberg corrected determined *P*_adj_ value of <0.05. For gene ontology (GO) analysis, DEGs were further filtered to be changed by at least 2-fold. GO analysis was performed using IDEP^24^ and Metascape.^25^ A gene list Venn diagram was generated using the online tool found at https://www.bioinformatics.org/gvenn/. Gene set variation analysis was performed by using Gene Set Variation Analysis (GSVA; Bioconductor) with gene signatures of interest from MSigDB. GSVA enrichment was performed using counts after variance-stabilizing transformation. Lastly, Gene Set Enrichment Analysis (GSEA) software was used for enrichment analysis of GBM subtypes. Input gene list files were generated with DESeq2. Three biological replicates were used for each experimental condition.

### Chromatin Immunoprecipitation-qPCR

Chromatin immunoprecipitation was performed according to the manufacturer’s instructions (Active Motif) with the following modifications. Chromatin was sheared in diluted lysis buffer to 200 to 500 bp using a Covaris M220 Focused Ultrasonicator with the following parameters: 3 min, peak incident power 75%, duty factor 10%, and 200 cycles per burst. ChIP-grade antibodies were obtained from commercially available sources: anti-BRD2 (CST, #5848), anti-RelA/p65 (CST, #8242), anti-BRD4 (CST, #13440). Five percent of the chromatin was not exposed to antibodies and was used as an input control. DNA quantity for each ChIP sample was normalized against input DNA.

### Quantitative Real-time PCR

RNA was extracted with the RNeasy Plus kit (Qiagen, #74134) according to the manufacturer’s instructions. Reverse transcription of mRNA was performed using 3-5 μg RNA with RNA to cDNA EcoDry Premix (Takara, #639549). For real-time PCR analysis, 1 μL of cDNA (10 ng of starting RNA) was amplified per reaction using the iTaq Universal SYBR Green Supermix (Bio-Rad, #1725124) and the Bio-Rad CFX96 qPCR system.

### shRNA-mediated Knockdown

For lentivirus production, 293T cells were transfected with BRD2 shRNA (Sigma), psPAX2, and pMD2.G packaging constructs using TransIT-VirusGEN (Mirus). Supernatants containing high-titer lentivirus were collected 48 h after transfection and filtered through a 0.45 µm cellulose acetate filter before use. Viral preparations were then purified by LentiX concentrator (Takara Bio). GSCs were infected for 24 h at 37 °C, and the supernatant containing the virus was replaced with fresh culture medium. Infected cells were selected by puromycin.

### Matrigel Invasion Assay

GSCs (1 × 10^5^ cells) were suspended in serum-free culture medium and seeded into 24-well Transwell inserts (8.0 µm). Medium with indicated factors was added to the remaining receiver wells. After 16-24 h, the invaded GSCs were fixed and stained with crystal violet (0.05%, Sigma) and counted as cells per field of view using a Keyence BZ-X700 microscope.

### GSC Irradiation and Treatment

Cells were irradiated using X-RAD 320 (Precision X-ray; 320 kV, 12.5 mA) with a single dose of either 2 or 4 Gy.

### Orthotopic Xenografts

Animal research experiments were conducted in accordance with the regulations of the UCSD Animal Care Program, protocol number S00192M. Female BALB/c nude mice (4-5 weeks old) were used for intracranial tumor implantation. A total of 1-2 × 10^5^ cells in a 3-5 μL volume was injected stereotactically into the brain (1.0 mm anterior and 2.0 mm right to the bregma, and 3 mm deep from the inner plate of the skull). Tumor growth was monitored using the FMT 2500 fluorescence tomography system (PerkinElmer). For drug treatment studies, mice received daily (5 days/week) oral gavage of vehicle or GSK620 at 30 mg/kg. The vehicle formulation consisted of 10% DMSO, 40% PEG, 5% Tween-80, and 45% saline. Mice were euthanized per institutional guidelines for animal welfare and experimental conduct.

### Clonogenic Assay

Cells were exposed to varying doses of IR (0-6 Gy) and then seeded at appropriate densities in 6-well plates. Following irradiation, cells were incubated for 14 days to allow colony formation. Colonies were then fixed with 4% formaldehyde and stained with 0.5% crystal violet. The surviving fraction was calculated by normalizing the plating efficiency of irradiated cells to that of non-irradiated control cells.

### Immunohistochemistry

The Histology Core Facility at UCSD Pathology prepared 5 µm formalin-fixed, paraffin-embedded (FFPE) tissue sections. Sections were immunostained for Human Nucleoli (clone NM95; Abcam; ab190710) and Iba-1 (Wako; 019-19741). See Supplementary Methods for details.

### Cell Growth Assay

Five replicates were plated per each condition in black-walled plate, clear-bottom 96-well plates. Cell growth was analyzed using an ATPlite 1step assay kit (PerkinElmer 6016731) following the manufacturer’s instructions.

### Statistical Analyses

All experiments were repeated at least 3 times. Statistical analyses were performed using GraphPad Prism 9. Data sets were analyzed using unpaired *t*-tests or multiple comparisons by one-way ANOVA or two-way ANOVA as appropriate. In figures, asterisks indicate *P < 0.05, **P < 0.01, and *** *P* < 0.001, ****P < 0.0001.

## Results

### PTEN Loss Drives MES Transition by Regulating the Chromatin Localization of RelA, BRD2, and BRD4 Proteins

**Figure 1.**
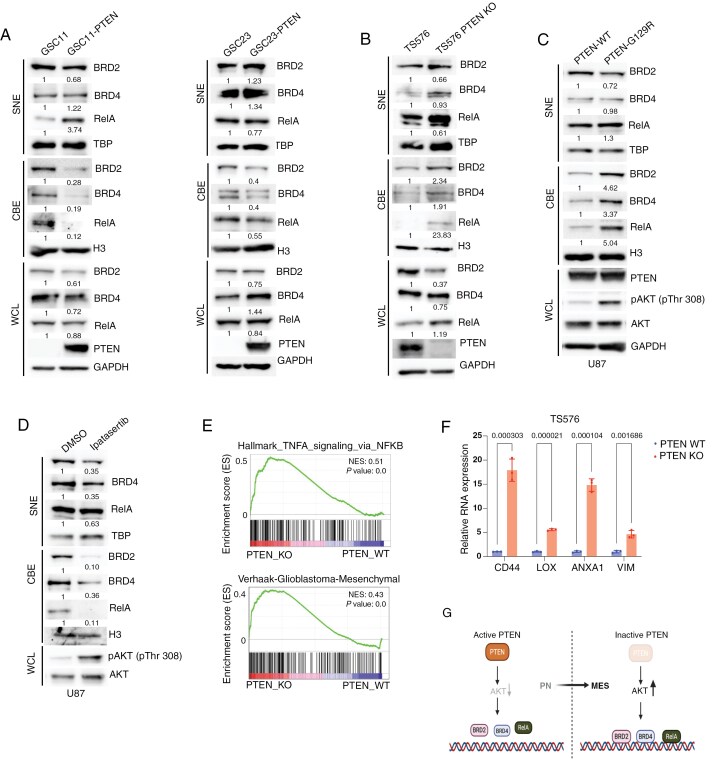
PTEN negatively regulates chromatin binding of BRD2, BRD4 and RelA. (A-D) Immunoblot analyses of BRD2, BRD4, and RelA in SNE, CBE, and WCL from: (A) GSC11 and GSC23 cells stably expressing either empty vector or wild-type PTEN. (B) TS576 cells with or without PTEN deletion; (C) U87 cells stably expressing wild-type (PTEN-WT) or phosphatase dead (PTEN-G129R) PTEN; (D) U87 cells treated with DMSO or Ipatasertib (1 µM, 24 h). Representative blots (*n* = 3). TBP, H3, and GAPDH serve as loading controls for SNE, CBE, and WCL fractions, respectively. (E) GSEA graphs from bulk RNA-seq in TS576 cells with (PTEN_WT) or without (PTEN_KO) PTEN expression. (F) qPCR analysis showing MES gene expression in TS576 cells with or without PTEN expression (*n* = 3, mean ± SD). (G) Schematic model illustrating PTEN-mediated regulation of MES transition in GBM.

We previously demonstrated that NF-κB-mediated recruitment of BRD4 promotes IL-6 transcription in heterogeneous GBM models.^22^ NF-κB activation is frequently observed in GBM and often coincides with activation of the PI3K-AKT signaling pathway.^12^ Given the central role of PTEN in regulating the PI3K-AKT signaling, we hypothesized that BET proteins may be regulated by this pathway in GBM. We overexpressed PTEN in PTEN-deficient GSC11, GSC23, and U87 cells, and assessed the chromatin binding of BRD2 and BRD4 by separating the nuclear lysate into soluble nuclear extract (SNE) and chromatin-bound (CB) fractions. PTEN expression resulted in a decreased association of BRD2 and BRD4 with chromatin, compared to WCL (whole cell lysate) and SNE fractions (Figure 1A and Supplementary Figure S1A). Conversely, PTEN knockout in TS576 cells increased the chromatin localization of BRD2 and BRD4 (Figure 1B). We also observed variable levels of BRD2, BRD4, and RelA in the WCL of different GSC lines, depending on PTEN status, indicating that while PTEN consistently modulates the chromatin association of these proteins, its effect on their overall stability is variable and cell line dependent. The phosphatase activity of PTEN is essential for its tumor-suppressive role and PI3K-AKT inhibition.^26^ Expression of the phosphatase-dead PTEN (G129R) mutant led to increased chromatin association of BRD2 and BRD4 compared to wild-type PTEN, indicating phosphatase-dependent regulation of BET protein activity (Figure 1C). Moreover, pharmacological inhibition of AKT with Ipatasertib decreased the chromatin localization of BRD2 and BRD4 in concert with the known feedback phosphorylation of Akt at T308^27^ (Figure 1D). Interestingly, RelA and BET proteins exhibited similar patterns of chromatin localization across all conditions (Figure 1A to D, Supplementary Figure S1A). These results suggest that PI3K-AKT activation downstream of PTEN loss enhances chromatin deposition of RelA, BRD2, and BRD4 proteins in GBM.

Given that NF-κB activation is a known driver of MES transition in GBM,^6^ we investigated whether PTEN loss-induced chromatin association of RelA, BRD2, and BRD4 contributes to this transcriptional reprogramming. RNA-seq on PTEN-deleted TS576 cells revealed significant enrichment of MES-associated gene signatures, NF-κB signaling pathways, and increased invasion compared to parental cells (Figure 1E and F, and Supplementary Figure S1B and S1C). Collectively, these results indicate that PTEN inactivation enhances the chromatin association of RelA and BET proteins, which, in turn, promotes a MES-like transition in GBM (Figure 1G).

### Acetylation of RelA at Lysine 310 Drives MES Transition in GBM

**Figure 2.**
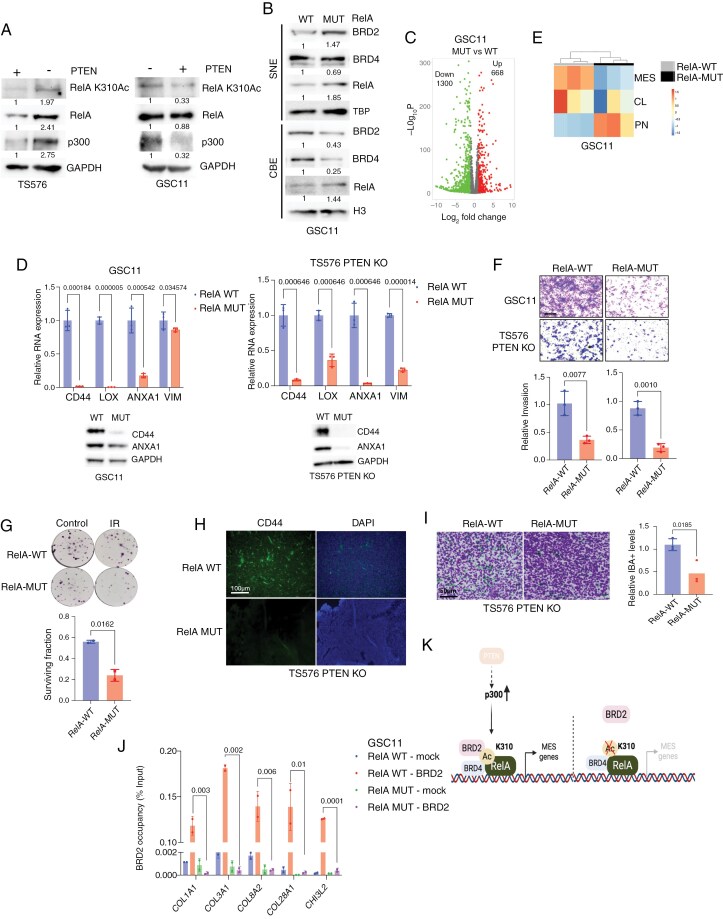
RelA Lysine 310 acetylation promotes MES transition. (A) Immunoblot analysis of acetylated RelA (K310Ac) and p300 in TS576 (left panel) and GSC11 (right panel) cells with or without PTEN expression. (B) Immunoblot analysis of indicated proteins in SNE and CBE fractions of GSC11 cells expressing either RelA-WT or RelA-MUT. Representative blots (*n* = 3). (C) Volcano plot showing differential gene expression in RelA-WT versus RelA-MUT expressing GSC11 cells. Data are presented as log₂ fold change versus −log₁₀ FDR. (D) q-PCR analysis of MES gene expression in GSC11 and TS576 PTEN KO cells expressing either RelA-WT or RelA-MUT (*n* = 3; mean ± SD). (E) Heatmap of GSVA scores for GSC11 cells expressing either RelA-WT or RelA-MUT, grouped by GBM subtype. (F) Matrigel invasion assay comparing RelA-WT and RelA-MUT expressing GSC11 and TS576 PTEN KO cells. Invasive cells were quantified across five random fields and expressed as relative percentages (*n* = 3; mean ± SD). Scale bar, 100 µm. (G) Clonogenic survival assay of TS576 PTEN KO cells expressing RelA-WT or RelA-MUT following IR (4 Gy). Cell colonies were analyzed 14 days after IR (*n* = 3; mean ± SD). (H) IF staining images of CD44 in the brains of patient-derived xenograft tumor-bearing mice derived from TS576 PTEN KO cells expressing RelA-WT or RelA-MUT. Scale bar, 100 μm. (I) Dual IHC staining of human glioma cells (NM95; purple) and IBA1 + macrophages/microglia (green) in mice GBM tumors from (H), scale bar, 50 µm. Quantification of IBA^+^ cells are shown (*n* = 3 per each group). (J) ChIP-qPCR analysis of BRD2 occupancy at MES gene promoters in GSC11 cells expressing either RelA-WT or RelA-MUT. Data represent fold enrichment over input (*n* = 3 biological replicates, each with 2 technical replicates; mean ± SD). (K) Schematic model the role of RelA acetylation in promoting MES gene expression in GBM.

RelA undergoes various posttranslational modifications that influence its stability, subcellular localization, and interactions with other proteins.^28^ Among these, acetylation at lysine 310 (K310) has been shown to promote the recruitment of BRD4 to super enhancers and promoters of NF-κB target genes, thereby activating transcription.^29^ We hypothesized that RelA K310 acetylation following PTEN loss facilitates the recruitment of BRD2 and BRD4 to chromatin via their bromodomains (BD1 and BD2), thereby promoting MES transition. Consistent with this, we observed elevated levels of RelA K310 acetylation in PTEN-deleted TS576, while re-expression of PTEN reduced acetylation in GSC11 and GSC23 (Figure 2A and Supplementary Figure S2A). Given that the histone acetyltransferase p300 has previously been implicated in mediating RelA acetylation at K310,^30^ we assessed its expression. We found that p300 levels were upregulated in PTEN-deleted cells and downregulated upon PTEN overexpression (Figure 2A). These findings suggest that increased p300 expression may contribute to enhanced RelA K310 acetylation in PTEN-deficient GBM cells. To delineate the functional role of this acetylation, we generated an endogenous knock-in mutation in the *RELA* gene by substituting lysine 310 with a nonacetylatable arginine (K310R), which is hereafter referred to as RelA-MUT (Supplementary Figure S2B). This mutation led to marked reduction in RelA K310 acetylation in both TS576 and GSC11 cell lines (Supplementary Figure S2C). Notably, the RelA mutation did not affect nuclear translocation in response to TNF-α, nor did it alter NF-κB reporter activation or its inhibition by a IκBα super-repressor (IκBαSR) (Supplementary Figure S2D and E). Notably, RelA-MUT cells showed a marked reduction in BRD2 and BRD4 chromatin binding relative to RelA-WT GSCs (Figure 2B), while chromatin localization of RelA increased. These findings suggest that K310 acetylated chromatin-bound RelA is required for efficient recruitment of BET proteins to chromatin. To define the transcriptional effects of RelA K310 acetylation, we performed RNA-seq on RelA-MUT and RelA-WT GSC11. Disruption of K310 acetylation led to significantly more downregulated (1300, 2-fold decrease, *P*_adj_ < 0.05) than upregulated (668, 2-fold increase, *P*_adj_ < 0.05) genes (Figure 2C). The GO analysis of the downregulated genes revealed a significant enrichment for matrisome-associated terms (Supplementary Figure S2F), which are functionally linked to the MES gene signature in GBM.^31,32^ To further investigate the link between K310 acetylation and MES identity, we assessed MES gene expression by q-PCR and immunoblotting. MES genes were consistently downregulated in RelA-MUT cells compared to WT controls (Figure 2D and Supplementary Figure S2G). GSVA demonstrated enrichment of the MES signature in RelA-WT cells, whereas RelA-MUT cells displayed enrichment for a PN signature (Figure 2E). Functionally, RelA-MUT cells exhibited reduced invasiveness (Figure 2F and Supplementary Figure S2H) and increased sensitivity to IR (Figure 2G), consistent with a less aggressive, non-MES phenotype (Figure 2G). To evaluate in vivo tumorigenic potential, we orthotopically implanted RelA-WT and RelA-MUT cells into nude mice. RelA-MUT GSC11 cells failed to form tumors (data not shown), while TS576 *PTEN* KO/RelA-MUT implanted mice displayed similar survival compared to RelA-WT tumors (Supplementary Figure S2I). This discrepancy may reflect intrinsic genetic differences between the 2 models. Nevertheless, staining of endpoint RelA-MUT tumors revealed reduced expression of the MES marker CD44 (Figure 2H). Furthermore, IBA1 staining showed decreased infiltration of TAMs, a feature previously associated with the MES phenotype in GBM^33^ (Figure 2I).

### RelA Lysine310 Acetylation is Essential for BRD2 Recruitment to MES Gene Promoters

To determine whether the downregulation of MES genes in RelA-MUT cells results from impaired recruitment of BET proteins to MES gene promoters, we performed ChIP-qPCR to assess the localization of RelA, BRD2, and BRD4 at a panel of extracellular matrix (ECM)-associated MES gene promoters. Both BRD2 and BRD4 were enriched at these promoters in RelA-WT cells (Figure 2J and Supplementary Figure S2J). In contrast, RelA-MUT cells exhibited a marked reduction in BRD2 occupancy, while BRD4 binding was only modestly affected (Figure 2J and Supplementary Figure S2J). Interestingly, RelA-MUT cells demonstrated increased RelA binding at these promoters despite diminished transcription of the corresponding genes (Supplementary Figure S2K). These observations support a model in which BRD2 deposition at RelA-bound regulatory elements is required for full transcriptional activation, particularly at MES gene loci where BRD4 may already be constitutively present to support basal gene expression.^34^ Together, these findings establish that RelA K310 acetylation is a key molecular event that facilitates BRD2 recruitment to MES gene promoters, thereby promoting their transcription in GBM (Figure 2K).

### BRD2 Promotes MES Transition in GBM

**Figure 3.**
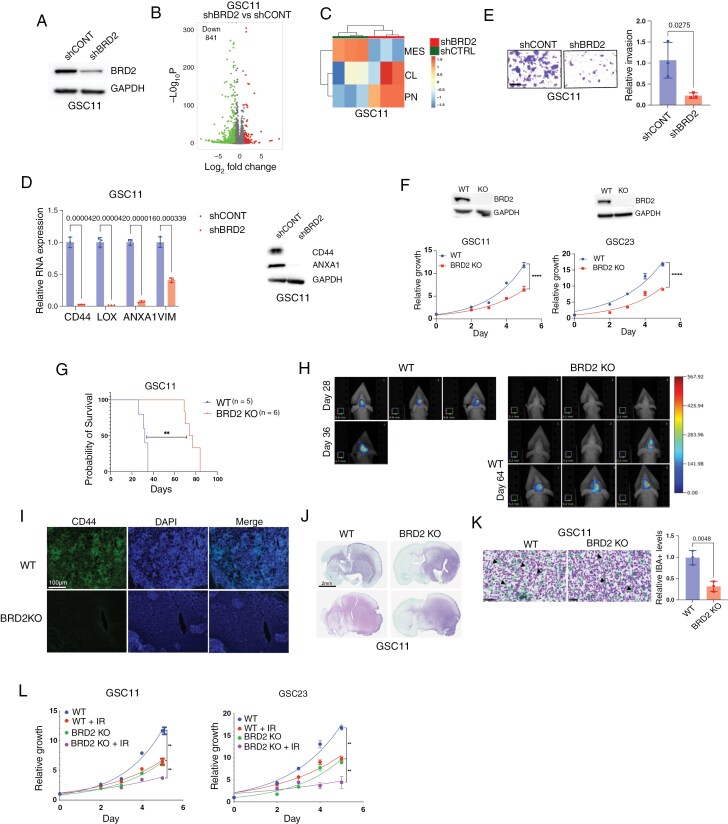
BRD2 promotes MES transition in GBM. (A) Immunoblot analysis of BRD2 in GSC11 with shCONT or shBRD2. (B) Volcano plot showing differential gene expression in shBRD2 versus shCONT expressing GSC11 cells. Data are presented as log₂ (fold change) versus −log₁₀(FDR). (C) Heatmap of GSVA scores for shBRD2 or ShCTRL expressing GSC11 cells, grouped by GBM subtype. (D) q-PCR analysis of MES gene expression in shBRD2 or shCONT expressing GSC11 cells (*n* = 3; mean ± SD). (E) Matrigel invasion assay comparing shCONT or shBRD2 expressing GSC11 cells. Invasive cells were quantified across 5 random fields and expressed as relative percentages (*n* = 3; mean ± SD). Scale bar, 100 µm. (F) (Top) Immunoblot showing BRD2 levels in GSC11 and GSC23 cells. (Bottom) ATPlite assay showing cell growth differences in BRD2 deleted GSC11 and GSC23 cells (*n* = 3 per group; mean ± SD). (G) Kaplan–Meier survival curves of mice bearing intracranial tumors derived from GSC11 with or without BRD2 deletion. (H) FMT imaging of orthotopic xenografts derived from iRFP720 expressing GSC11 cell with or without BRD2 expression (*n* = 3 per group). (I) IF staining images of CD44 in the brains of patient-derived xenograft tumor-bearing mice derived from (G). Scale bar, 100 μm. (J) IHC showing in vivo growth of GSC11 cells with or without BRD2 expression. Glioma cells (NM95; purple). Scale bar, 2 mm. (K) Dual IHC staining of human glioma cells (NM95; purple) and IBA1 + macrophages/microglia (green) in mice GBM tumors from (G), scale bar, 50µm. Quantification of IBA^+^ cells are shown (*n* = 3 per each). (L) Cell proliferation assay with ATPlite showing cell growth differences in GSC11 and GSC23 cells with or without BRD2 expression post-IR (4 Gy) (*n* = 3 per group; mean ± SD).

We performed BRD2 knockdown to evaluate its role in MES transition and observed a significant reduction in ECM gene expression by RT-qPCR (Figure 3A and Supplementary Figure S3A). Transcriptomic profiling using RNA-seq revealed 841 genes that were significantly downregulated in BRD2-depleted cells compared to controls (Figure 3B). GO analysis of these downregulated genes revealed enrichment for matrisome-associated terms and those related to the MES phenotype, which was overrepresented among the top pathways (Supplementary Figure S3B). Notably, 213 genes were commonly downregulated in both RelA-MUT and BRD2 knockdown conditions (Supplementary Figure S3C). GO analysis of these shared targets identified matrisome-associated terms among the top pathways (Supplementary Figure S3D), suggesting that BRD2 and RelA co-regulate MES gene expression. GSVA and GSEA demonstrated a loss of the MES subtype signature and concurrent enrichment of the CL and PN subtype signatures in BRD2-depleted cells (Figure 3C and D and Supplementary Figure S3E), supporting a role for BRD2 in promoting the PN-to-MES transition. Previous studies have identified the AP-1 transcriptional factor complex as a key driver of MES gene expression in GBM.^9^ Consistent with this, GSEA revealed reduced expression of AP-1 target genes in BRD2 knockdown cells (Supplementary Figure S3F), suggesting BRD2 acts as a co-regulator of transcription factors involved in MES transition. To further support our findings, we analyzed an independent, published RNA-seq dataset^35^ from GSCs and confirmed a reduction in MES gene expression in BRD2-depleted cells (Supplementary Figure S3G). BRD2 knockdown also downregulated NF-κB pathway genes (Supplementary Figure S3H) and impaired cellular invasiveness (Figure 3E and Supplementary Figure S3I).

Next, we generated BRD2 knockout GSC11 and GSC23 lines (Figure 3F). BRD2 KO cells displayed reduced proliferation, consistent with the known role of BRD2 in cyclin regulation^36^ (Figure 3F). BRD2 KO impaired the upregulation of MES signature genes following TNF-α induced NF-κB activation (Supplementary Figure S3J), reinforcing its role in NF-κB dependent MES gene regulation. Orthotopic implantation of BRD2 KO cells into immunocompromised mice resulted in significantly prolonged survival compared to controls (Figure 3G and H). Immunofluorescence (IF) analysis of endpoint tumors revealed decreased expression of the MES marker CD44, diminished tumor invasion, and a lower abundance of TAMs in BRD2 KO tumors (Figure 3I to K), consistent with a suppressed MES phenotype. Notably, BRD2 KO tumors exhibited increased Ki67 staining, indicative of an enrichment in proliferative, PN-like tumor cells^9^ (Supplementary Figure S3K). Additionally, BRD2-deficient cells exhibited enhanced sensitivity to both IR and temozolomide (TMZ) (Figure 3L and Supplementary Figure S3L). Collectively, these findings identify BRD2 as a critical regulator of MES transition in GBM and its loss promotes a proliferative PN-like state, enhancing sensitivity to standard therapies.

### BRD2 Bromodomains are Essential for MES Transition

**Figure 4.**
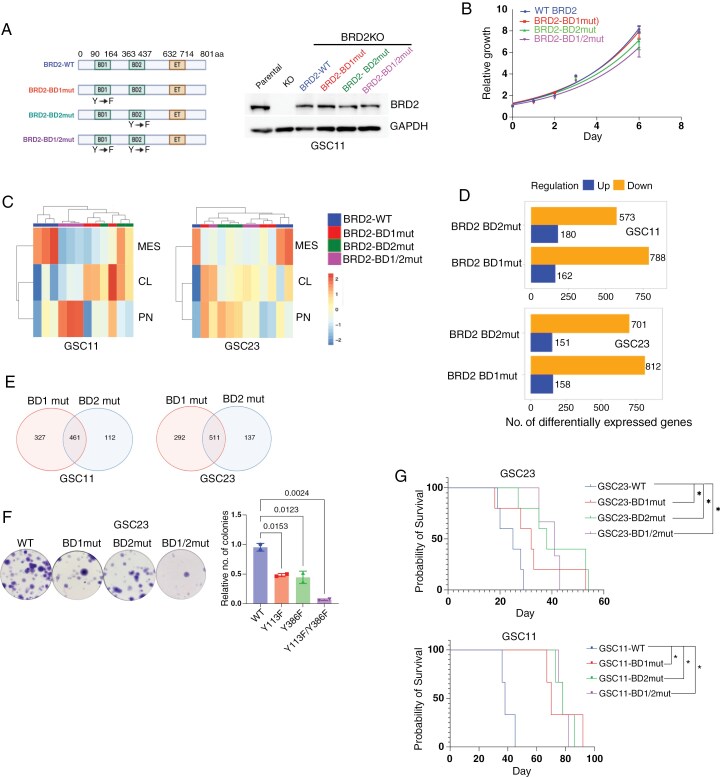
GBM cell MES transition is mediated by BRD2 bromodomains. (A) Schematic illustration of the domains of BRD2 (left). IB analysis of BRD2 deleted GSC11 cells rescued with BRD2-WT (wildt-ype), BRD2-BD1mut (Y113F), BRD2-BD2mut (Y386F), and BRD2-BD1/2mut (Y113F/Y386F). (B) ATPlite assay showing cell growth differences in GSC11 cells expressing BRD2-BD mutants (*n* = 3 per group; mean ± SD). One-way ANOVA with Dunnett’s multiple-comparison test. (C) Heatmap of GSVA scores for GSC11 cells GSC11 and GSC23 cells expressing BRD2–BD mutants, grouped by GBM subtype. (D) Bar plots showing gene expression differences in GSC11 and GSC23 cells expressing BRD2–BD mutants. (E) Venn diagram showing shared genes that are commonly downregulated in GSC11 and GSC23 expressing BRD2-BD1 vs BRD2-BD2 mutants. (F) Clonogenic assay with GSC23 cells expressing BRD2-BD mutants (*n* = 3; mean ± SD). (G) Kaplan–Meier survival curves of mice bearing intracranial tumors derived from GSC11 (*n* = 3) and GSC23 (*n* = 5) expressing BRD2-WT or BRD2-BD mutants.

BRD2 contains 2 N-terminal bromodomains, BD1 and BD2, which recognize and bind to acetyl-lysine residues on histones and other regulatory proteins.^14^ To elucidate the role of these bromodomains in regulating the MES phenotype, we re-expressed either wild-type BRD2 (BRD2-WT), or bromodomain-mutant variants-BRD2-Y113F (BD1 mutant), BRD2-Y386F (BD2 mutant), and BRD2- Y113F/Y386F (double mutant)^36^ in BRD2 knockout GSC11 and GSC23 cells (Figure 4A and Supplementary Figure S4A). In GSC11, re-expression of either wild-type or mutant BRD2 had no significant effect on proliferation (Figure 4B). However, RNA-seq followed by GSVA enrichment analysis for Verhaak GBM subtypes revealed that mutation of either BD1, BD2, or both resulted in loss of the MES signature (Figure 4C and Supplementary Figure S4D). Notably, disruption of BD1 or BD2 individually led to enrichment of the CL subtype signature, while the mutation of both bromodomains resulted in enrichment of the PN subtype signature (Figure 4C and Supplementary Figure S4D). These results demonstrate that both BD1 and BD2 are necessary for the maintenance of CL and MES transcriptional programs in GSC11. In contrast, in GSC23 cells, expression of BRD2 bromodomain mutants led to increased proliferation relative to BRD2-WT (Supplementary Figure S4B). RNA-seq analysis indicated that these cells exhibited reduced MES gene expression and increased expression of hallmark G2/M checkpoint genes, consistent with elevated cell cycle activity (Figure 4C and Supplementary Figure S4E). Importantly, MES gene expression in both GSC11 and GSC23 was dependent on intact BRD2 bromodomains. Inhibition of either domain was sufficient to downregulate the MES subtype signature. Disruption of BD1 resulted in more downregulated genes than BD2 (Figure 4D), with substantial overlap between gene sets co-regulated by both bromodomains (Figure 4E). Functionally, cells expressing BD1, BD2, or double mutants showed a significant reduction in colony numbers, with the BD1/BD2 double mutant having the most pronounced effect (Figure 4F). In vivo, orthotopic implantation of GSCs expressing BRD2 BD mutants in mice resulted in prolonged survival compared to those implanted with BRD2-WT cells (Figure 4G). Collectively, these data underscore the essential role of BRD2 bromodomains in maintaining the MES state in GBM. Disruption of either BD1 or BD2 is sufficient to impair MES gene expression, reduce tumorigenicity, and improve survival in vivo, highlighting BRD2 bromodomain inhibition as a potential therapeutic strategy for MES-subtype GBM.

### BET-BD2 Inhibitors Downregulate MES Gene Expression in GBM

**Figure 5.**
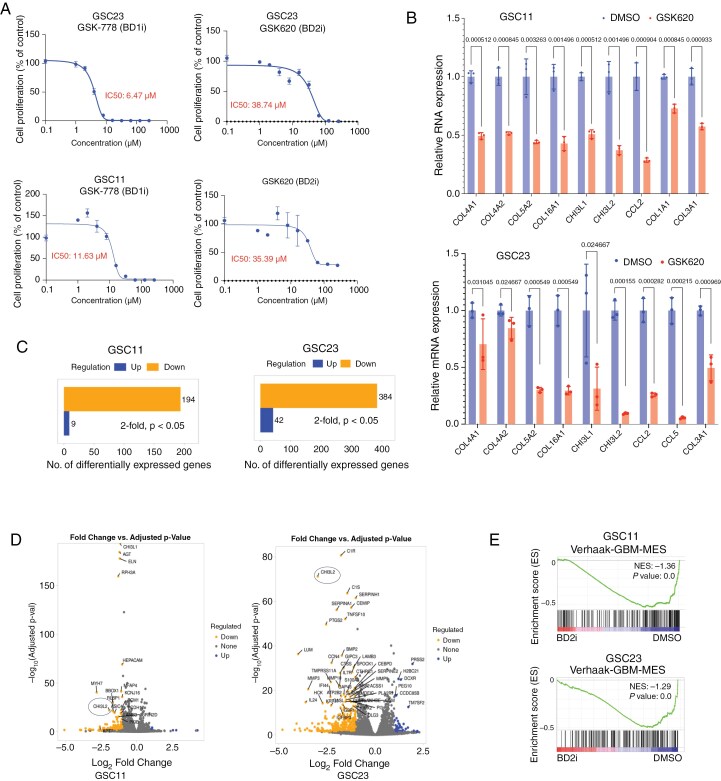
BET-BD2 inhibitors downregulate MES gene expression. (A) IC50 assays with ATPlite showing cell proliferation in GSCs treated with varying doses of BD1 inhibitor (GSK778) or BD2 inhibitor (GSK620) (*n* = 3). (B) qPCR analysis of MES gene expression in GSC11 and GSC23 cells treated with GSK620 (0.5-5 µM, 24-48 h). (C) Bar plots showing gene expression differences in GSC11 and GSC23 cells treated with GSK620 (0.5-5 µM, 24-48 h). (D) Volcano plot showing differential gene expression in GSCs treated with GSK620 (0.5-5 µM, 24-48 h). Data are presented as log₂ (fold change) versus −log₁₀ (FDR). (E) GSEA graphs from bulk RNAseq for GSCs treated with GSK620 or DMSO (0.5-5 µM, 24-48 h).

BET inhibitors have shown potent antitumor activity in preclinical studies.^37^ However, their clinical translation has been hindered by significant toxicity.^38^ Recently developed BD2-selective inhibitors demonstrated reduced toxicity compared to pan-BET or BD1-selective inhibitors.^34,39^ Our results indicate that disruption of BRD2–BD2 is sufficient to downregulate MES gene expression and extend survival in GBM-bearing mice. Based on this, we hypothesized that BD2-selective inhibition may effectively suppress MES gene expression while minimizing cytotoxic effects relative to BD1 or pan-BET inhibitors. Our IC50 analysis across multiple GSC lines found that the BD2-selective inhibitor (GSK620) exhibited higher IC50 values than the BD1 inhibitor (GSK778) (Figure 5A and Supplementary Figure S5A), consistent with the lower cytotoxicity profile previously reported for BD2-specific agents.^34^ Furthermore, treatment of GSCs with 2 BD2-selective inhibitors (GSK620 and ABBV-744) resulted in significant downregulation of many MES-associated genes, as assessed by RT-qPCR (Figures 5B and Supplementary Figure S5B). We next performed RNA-seq on GSC11 and GSC23 cells treated with GSK620 and identified DEGs( ≥ 2-fold change, *padj* < 0.05). GSK620 treatment downregulated 194 genes in GSC11 and 384 genes in GSC23 (Figure 5C). Notably, *CHI3L2*, a key MES marker implicated in immune cell infiltration in glioma^^10^^, was among the genes commonly downregulated in both lines (Figure 5D). GSEA further confirmed a significant loss of MES gene signatures following GSK620 treatment in both GSCs and in a mouse MES GBM model^40^ (Figure 5E and Supplementary Figure S5C). BD2 inhibition also suppressed transcriptional programs associated with AP-1 and NF-κB signaling pathways (Supplementary Figure S5D). Together, these results demonstrate that BD2-selective BET inhibitors effectively downregulate MES gene expression in GBM models, supporting their translational promise as a therapeutic strategy for targeting the MES subtype of GBM.

### GSK620 is a Brain-Penetrant BET-BD2 Inhibitor that Extends Survival in GBM Models and Sensitizes GSCs to Therapy

**Figure 6:**
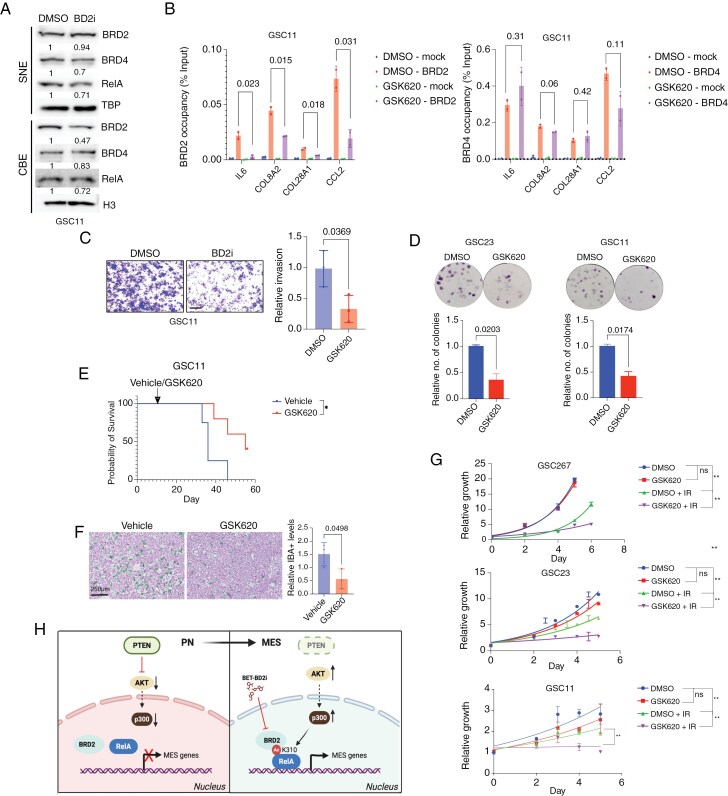
BET-BD2 inhibitors suppress MES phenotype, prolong survival, and sensitizes GSCs to IR. (A) IB analysis of indicated proteins in SNE and CBE fractions of GSC11 cells treated with DMSO or GSK620 (1 µM, 24 h). Representative blots (*n* = 3). (B) ChIP-qPCR analysis of BRD2 and BRD4 occupancy at MES gene promoters in GSC11 cells treated DMSO or GSK620 (1 µM, 24 h). Data represent fold enrichment over input (*n* = 3 biological replicates, each with 2 technical replicates; mean ± SD). **C)** Matrigel invasion assay comparing GSC11 cells treated with DMSO or GSK620 (0.5 µM, 24 h). Invasive cells were quantified across 5 random fields and expressed as relative percentage (*n* = 3; mean ± SD). Scale bar, 100 µm. (D) Clonogenic assay with for GSCs treated with DMSO or GSK620 (0.5-5 µM, 24 h). Data represents mean ± SD. (E) Kaplan–Meier survival curves for mice bearing intracranial tumors derived from GSC11 after treatments with vehicle or GSK620 (*n* = 5). (F) Dual IHC staining of human glioma cells (NM95; purple) and IBA1 + macrophages/microglia (green) in mice GBM tumors derived from (E), scale bar: 250 µm. Quantification of IBA^+^ cells are shown (*n* = 3 per each). (G) ATPlite assay of GSCs treated with DMSO or GSK620 (0.5-5 µM, 24 h) were exposed to 1 dose of IR (2-4 Gy) and assessed for cell growth on indicated timepoints (*n* = 3 per group; mean ± SD). (H) Schematic model illustrating RelA/BRD2 mediated regulation of MES transition in GBM. Created in BioRender. Vadla, R. (2025) https://BioRender.com/2alntun

To assess the effect of GSK620 on the chromatin localization of the BET proteins, we treated GSC11 cells with the BD2-selective inhibitor GSK620 and performed nuclear fractionation as described before (Figure 1). Treatment with GSK620 led to a more pronounced reduction in chromatin-bound BRD2, compared to BRD4 or RelA (Figure 6A), suggesting that BD2 inhibitor preferentially affects BRD2 chromatin binding. Consistently, ChIP-qPCR analysis revealed decreased BRD2 occupancy at MES gene promoters following GSK620 treatment, whereas BRD4 levels at these loci remained unchanged (Figure 6B). These findings suggest that the BD2 domain of BRD2 is essential for its recruitment to MES gene regulatory regions. Functionally, BD2 inhibition reduced GSC invasiveness (Figure 6C and Supplementary Figure S6A), and significantly impaired clonogenic survival (Figure 6D and Supplementary Figure S6B).

To evaluate the pharmacokinetic properties of GSK620 and its brain penetrance, we conducted in vivo analysis in mice treated with oral GSK620.^34^ The compound exhibited a brain-to-plasma area under the curve (AUC_0-24h_) ratio of 27.2% and a plasma half-life (*t*½) of 1.05 h (Supplementary Figure S6C), comparable to the kinetics of TMZ^41^ and indicating sufficient CNS penetration. To assess therapeutic efficacy in vivo, we orthotopically implanted GSC11 cells into mice and initiated GSK620 treatment after tumor establishment^21^ (Supplementary Figure S6D). GSK620-treated mice lived significantly longer than vehicle-treated controls (Figure 6E). IHC staining showed reduced infiltration of TAMs (Figure 6F). Given the well-documented resistance of MES GBM cells to standard therapies,^5^ we tested whether BD2 inhibition sensitizes GSCs to IR. Pretreatment with GSK620 followed by IR significantly decreased GSC proliferation compared to either treatment alone (Figure 6G). Similar radiosensitization was observed with another BD2 inhibitor, ABBV-744 (Supplementary Figure S6E). Notably, GSK620 did not impair the proliferation of neural progenitor cells (NPC) at the doses that sensitized GSCs to IR, indicating a favorable therapeutic index (Supplementary Figure S6F). Together, these findings establish GSK620 as a brain-penetrant, BD2-selective BET inhibitor that downregulates MES gene expression, suppresses tumor aggressiveness, extends survival in GBM models, and enhances the sensitivity of GSCs to radiotherapy.

## Discussion

In this study, we elucidated a critical role of BRD2 and its bromodomains in regulating GBM MES transition. Using CRISPR-mediated editing of the endogenous RELA gene in GSCs, we demonstrate that acetylation of RelA at lysine 310 recruits BRD2 to the promoters of MES genes, thereby driving their expression. BRD2 depletion abrogates the MES-like state and sensitizes GBM cells to standard therapies, specifically IR. Domain-specific mutagenesis revealed that both BD1 and BD2 are essential for maintaining the MES state, with loss of either shifting cells toward other transcriptional subtypes. Pharmacologic inhibition with BD2-selective BET inhibitors effectively downregulated MES gene expression, enhanced therapy response, and extended survival in GBM-bearing mice (Figure 6H).

The role of BET proteins in GBM is well established, with BRD4 being the most extensively studied member.^14^ BRD4 regulates key oncogenic programs including MYC, NOTCH1, NF-κB, and cell cycle genes, supporting cell proliferation, stemness, and therapy resistance.^42–44^ Its elevated expression in high-grade gliomas, success in other cancers, and correlation with poor prognosis have made it a focus of therapeutic efforts in GBM. However, the essentiality of BRD4 in both tumor and normal cells posed a significant barrier to clinical translation, as pan-BET inhibitors led to adverse toxicities in clinical trials.^45^ Although BRD2 has also been shown to be expressed at high levels in GBM,^16^ its role and function have not been well characterized.^35^

In our study, we observed increased chromatin association of both BRD2 and BRD4 upon PTEN loss, suggesting that BRD4 may be involved in MES transition in GBM. Indeed, BRD4 plays a crucial role in EMT in other cancers,^17^ suggesting a similar role in GBM. We were unable to generate homozygous BRD4 knockout clones in GSCs due to its essential role in cell viability, but BRD2 deletion was tolerated and led to a delay in cell growth along with suppression of MES gene expression. These findings suggest that BRD2 plays a key role in maintaining tumor cell plasticity and adaptive potential, unlike the essential survival gene BRD4. These observations prompted us to focus on BRD2, a comparatively less-characterized member of the BET family in GBM. Our data demonstrate that BRD2 loss disrupts MES transition and impairs tumor progression. BRD2 depletion also led to downregulated AP-1 gene signatures associated with MES transition,^9^ suggesting that BRD2 might be a common epigenetic modulator for multiple MES-promoting pathways in GBM. Importantly, we show that targeting BRD2 using a BD2-selective inhibitor sensitizes GBM cells to radiation therapy. This effect may be linked to impaired DNA damage response due to BET inhibition.^46^

IDH-WT gliomas consistently exhibit a limited set of epigenetically defined cell states, including OPC/NPC-like (collectively referred as PN), AC-like, and MES-like that reflect shared adaptive programs.^1^ These states differ in their response to therapy, with PN-like cells being more sensitive and MES-like cells more resistant. The concept of “state-selective lethality” proposes trapping GBM cells in a therapeutically vulnerable state, such as the PN, to enhance treatment efficacy.^47^ Our findings show that inhibition of RelA acetylation, BRD2 depletion, or treatment with BD2-selective inhibitors downregulates the MES-like state, promotes a shift toward CL/PN-like states, and sensitizes GSCs to IR. While we tested radiosensitization in vitro, future studies will be necessary to determine whether combining radiation with MES state inhibition using BD2 inhibitors results in a synergistic survival benefit. Notably, even in the absence of radiation, disruption of the MES state through BRD2 deletion, bromodomain mutation, or BD2 inhibition significantly prolonged survival in GBM models, suggesting that MES suppression alone can improve outcomes. The use of BD2-selective BET inhibitors is particularly relevant, as many BET-targeting compounds have already advanced into clinical trials,^37^ highlighting the translational potential of this strategy for targeting MES cell state in GBM. In GSC11 cells, mutation of either BRD2 bromodomains led to a transition away from the MES state toward a CL state, a shift not observed in GSC23 cells. We reasoned that this difference may be attributed to EGFR amplification in GSC11,^48^ as EGFR signaling is known to promote the CL cell-state. These findings suggest that underlying genetics, such as EGFR amplification, may influence the transcriptional programs adopted by GSCs once the MES state is attenuated. Based on this, it would be interesting to explore whether combining BD2 and EGFR inhibitors could be effective in EGFR-amplified GBMs and/or drive a further shift toward a more therapeutically vulnerable PN-like state.

Recent studies have shown that IR induces significant microenvironmental changes in both GBM patients and mice models, with increased deposition of ECM, TAM accumulation, and MES transition.^49,50^ Notably, interactions between immune cells and tumor cells have been implicated in promoting MES transition and tumor aggressiveness.^33^ In our study, BRD2 mutant GSC11 and GSC23 cells exhibited similar or even higher cell proliferation rates in vitro yet formed significantly slower growing tumors in vivo. This suggests that the tumor microenvironment, particularly the immune cell component or MES-associated ECM remodeling, may play a crucial role in driving tumor aggressiveness. Moreover, it suggests that these components may be attenuated in BRD2 mutant tumors. Given that IR also enhances ECM deposition, our findings suggest that combining BRD2 bromodomain targeting with BD2-selective inhibitors and standard therapies may help suppress ECM remodeling and prevent aggressive tumor recurrence.

Our findings position BRD2 as a novel, underexplored epigenetic vulnerability in GBM. Unlike pan-BET inhibitors, which have dose-limiting toxicities, we demonstrate that BD2-selective inhibition (BD2i) is less toxic and effectively sensitizes GBM cells to IR. Thus, targeting BRD2, either genetically or pharmacologically, represents a rational and potentially safer strategy to modulate GBM cell state plasticity and improve responsiveness to conventional therapies.

## Supplementary material

Supplementary material is available online at *Neuro-Oncology* (https://academic.oup.com/neuro-oncology).

noaf169_Supplementary_Data

noaf169_Supplementary_Figures_S1-S6

## Data Availability

Data have been deposited at Gene Expression Omnibus. Bulk RNA-seq data are available under accession number GSE304029.
