# Supplementary material for: BRD2 bromodomain-mediated regulation of cell state plasticity modulates therapy response in glioblastoma
Source: Neuro Oncol. 2025 Jul 19;27(11):2828–42. doi: 10.1093/neuonc/noaf169 (PMC12700489; doi:10.1093/neuonc/noaf169)
Supplement: noaf169_Supplementary_Figures_S1-S6 [file noaf169_supplementary_figures_s1-s6.docx]

**BRD2 Bromodomain-Mediated Regulation of Cell State Plasticity Modulates Therapy Response in Glioblastoma**

Raghavendra Vadla^1^, Brett Taylor^1,2^, Yohei Miyake^1^, Benjamin Lin^3,4^, Daisuke Kawauchi^1^, Shunichiro Miki^1^, Nidhi Nathwani^1^, Brandon M Jones^1^, Yashpreet Kaur^1^, Abhinaba Banerjee^5^, Philip Pham^1^, Jonathan Tsang^6^, Albert Baldwin^7^, David A. Nathanson^5^, Donald P. Pizzo^8^, C. Ryan Miller^3^, Frank B. Furnari^1^

^1^ Division of Regenerative Medicine, Department of Medicine, University of California San Diego, La Jolla, CA 92093, USA.
^2^ Medical Scientist Training Program, University of California, San Diego, La Jolla, CA 92093, USA.

^3^ Department of Pathology, Division of Neuropathology, Heersink School of Medicine, University of Alabama at Birmingham, Birmingham, AL 35294, USA.

^4^ Medical Scientist Training Program, University of Alabama at Birmingham, Birmingham, AL 35294, USA.

^5^ Department of Bioengineering, University of California, San Diego, La Jolla, CA 92093, USA.
^6^ Departments of Molecular and Medical Pharmacology, University of California, Los Angeles, CA 90095, USA.

^7^ UNC Lineberger Comprehensive Cancer Center, University of North Carolina School of Medicine, Chapel Hill, NC  27599, USA.

^8^ Department of Pathology, University of California San Diego, La Jolla, CA 92093, USA

**Running title:** BRD2 regulates GBM cell state plasticity

**Corresponding author**:

Frank Furnari, Ph.D.

Professor

University of California-San Diego 
Sanford Consortium for Regenerative Medicine

2880 Torrey Pines Scenic Drive

La Jolla CA 92037

858-534-7819

**Supplemental Figure 1**


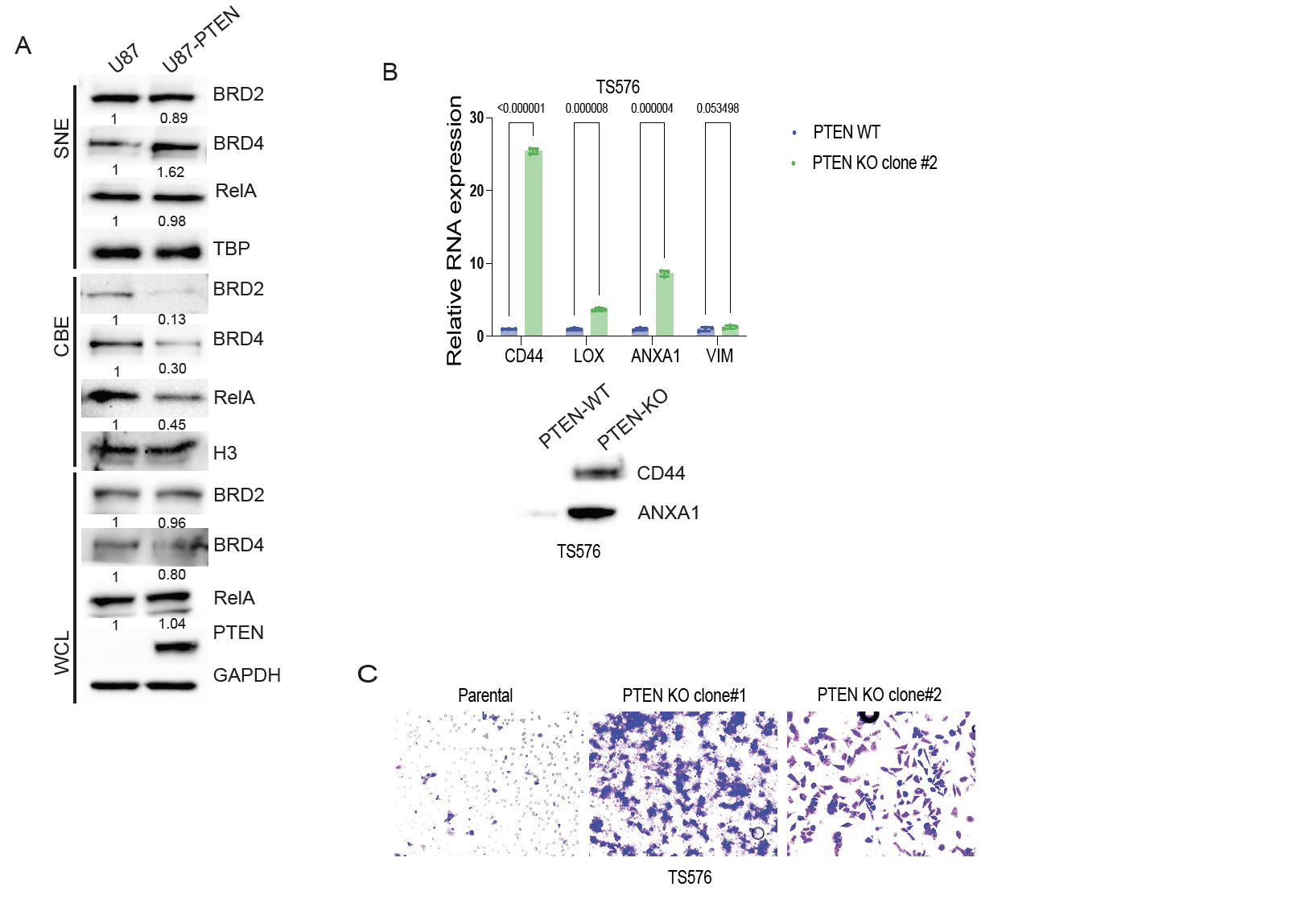


**Figure S1**. **A)** Immunoblot analyses of BRD2, BRD4 and RelA in soluble nuclear extract (SNE), chromatin-bound extract (CBE) and whole cell lysates (WCL) from U87 cells expressing either empty vector or wild-type PTEN. **B)** qPCR analysis and immunoblot showing MES gene expression in TS576 cells with or without PTEN expression (n=3 per group). **C)** Matrigel invasion assay comparing TS576 and TS576 PTEN KO cells (n = 3). Scale bar, 100μm.

**Supplemental Figure 2**

**
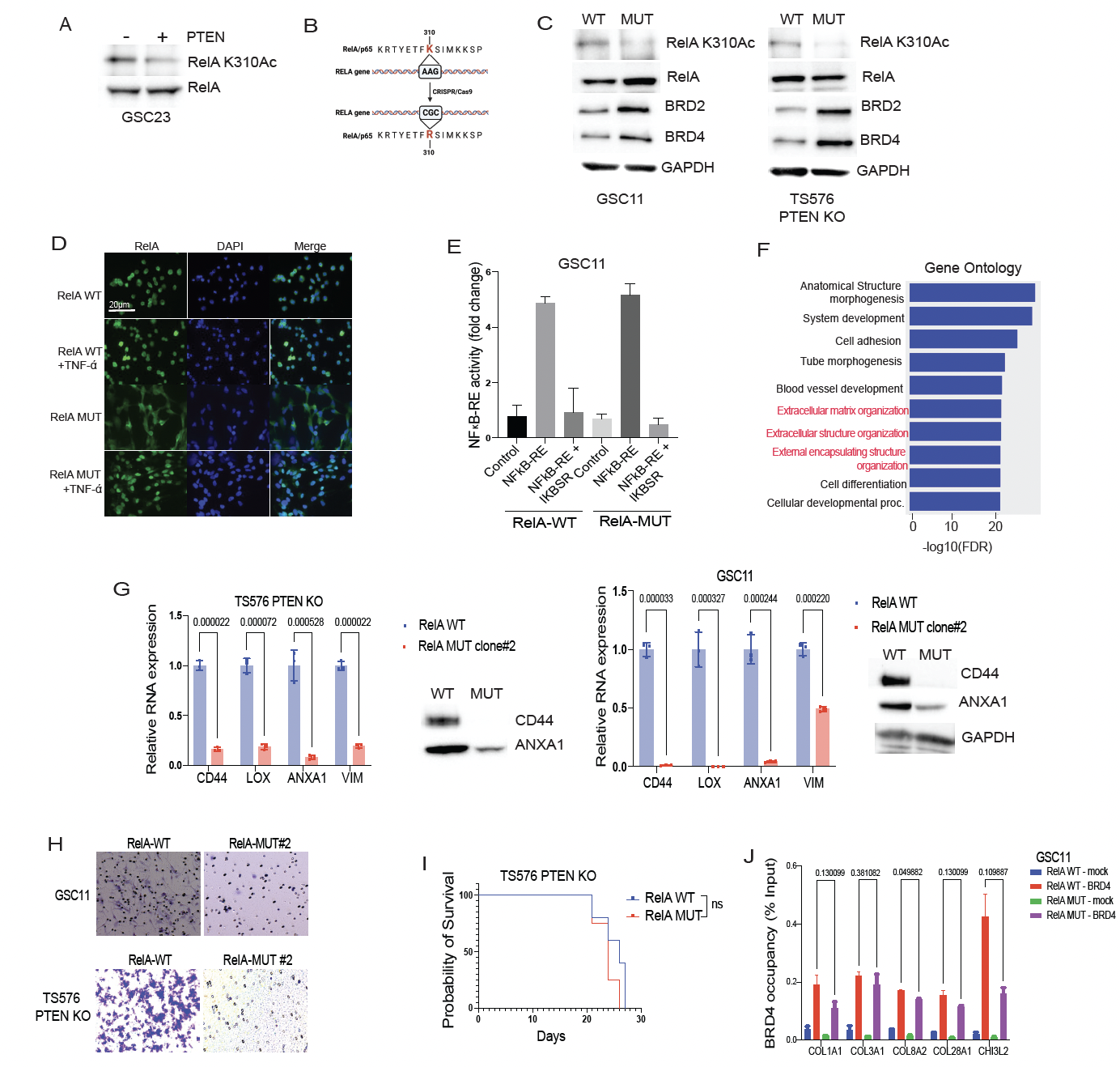
**

**
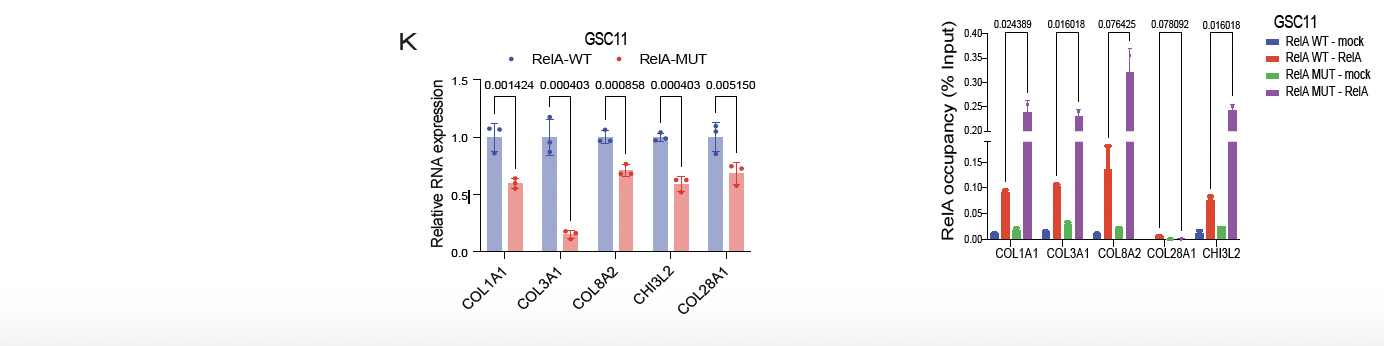
**

**Figure S2**. **A)** Immunoblot analysis of acetylated RelA (K310Ac) and p300 in GSC23 cells with or without PTEN expression. **B)** Schematic illustration for CRISPR/Cas9 knock-in mutation of *RELA* gene in GSCs. **C)** Immunoblot analysis of indicated proteins in WCLs of GSC11 and TS576 PTEN KO expressing either RelA-WT or RelA-MUT. **D)** IF images of RelA nuclear translocation in response to DMSO or TNF-α (20ng/ml, 20 min) in RelA-WT and Rel-MUT GSC11 cells. Scale bar indicates 20uM. **E)** Luciferase assay for NF-κB activity in RelA-WT or RelA-MUT expressing GSC11 cells with or without IKBSR expression**.** (n=3, mean ± SD). **F)** GO analysis of downregulated genes in GSC11 cells expressing RelA-MUT cells compared to RelA-WT. **G)** q-PCR analysis of MES gene expression in GSC11 and TS576 PTEN KO cells expressing either RelA-WT or RelA-MUT (n=3; mean ± SD). **H)** Matrigel invasion assay comparing GSC11 and TS576 cells expressing either RelA-WT or RelA-MUT (n = 3). Scale bar, 100μm. **I)** Kaplan-Meier survival curves of mice bearing intracranial tumors derived from TS576 PTEN KO expressing RelA-WT or RelA-MUT. **J)** ChIP-qPCR analysis of BRD4 and RelA occupancy at MES gene promoters in GSC11 cells expressing either RelA-WT or RelA-MUT. Data represent fold enrichment over input (n = 3 biological replicates, each with two technical replicates; mean ± SD). **K)** qPCR analysis showing ECM gene expression in GSC11 cells expressing either RelA-WT or RelA-MUT (n=3, mean ± SD).

**Supplemental Figure 3**

**
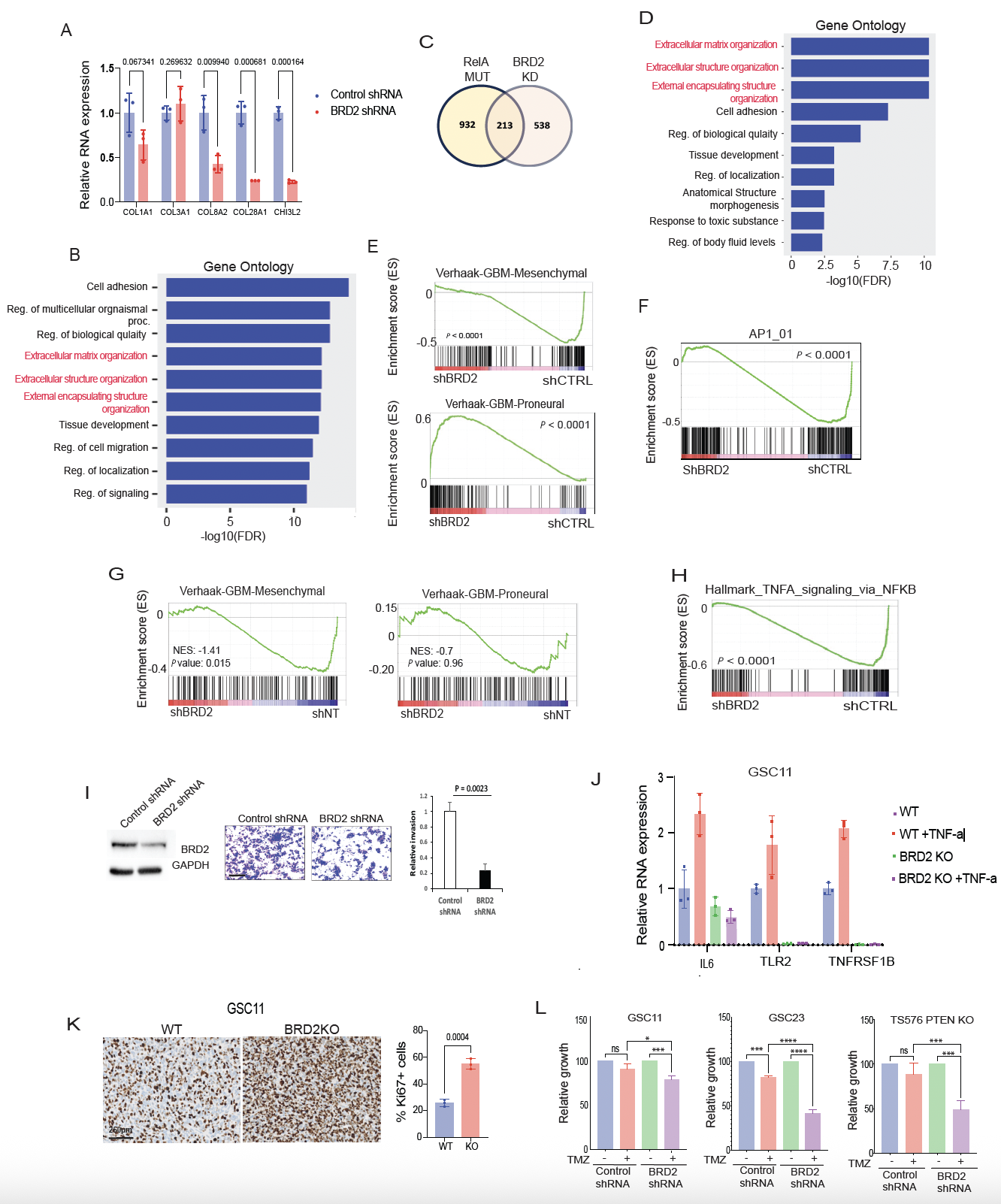
**

**Figure S3**. **A)** qPCR analysis showing ECM gene expression in shCONT and shBRD2 GSC11 cells. (n=3, mean ± SD). **B)** GO analysis of downregulated genes in GSC11 cells shBRD2 compared to shCONT. **C)** Venn diagram showing overlap of downregulated genes in GSC11 cells expressing RelA-MUT and those with shBRD2. **D)** GO analysis of shared downregulated genes in (C). **E, F)** GSEA graphs from bulk RNAseq in GSC11 cells with shCONT or shBRD2 expression. **G)** GSEA graphs from bulk RNAseq in GSCs cells with shNT or shBRD2 expression. **H)** GSEA graphs from bulk RNAseq in GSC11 cells with shCONT or shBRD2 expression. **I)** IB analysis showing BRD2 levels in shCONT and shBRD2 expressing TS576 PTEN KO cells (left). Matrigel invasion assay (right). Invasive cells were quantified across five random fields and expressed as relative percentage (n = 3; mean ± SD). Scale bar, 100μm. **J)** qPCR analysis of MES gene expression in GSC11 cells with or without BRD2 deletion, following TNF-α stimulation (20 min, 10 ng/mL). (n=3, mean ± SD). **K)** IHC staining of Ki67 in mice GBM tumors from **(**Figure 3J**)**, scale bar, 250μm. Quantification of Ki67^+^ cells (%) are shown (n=3, mean ± SD). **L)** ATPlite assay of shCONT or shBRD2 expressing GSCs were treated with TMZ (50uM, 24 h). (n=3, mean ± SD).

**Supplemental Figure 4**


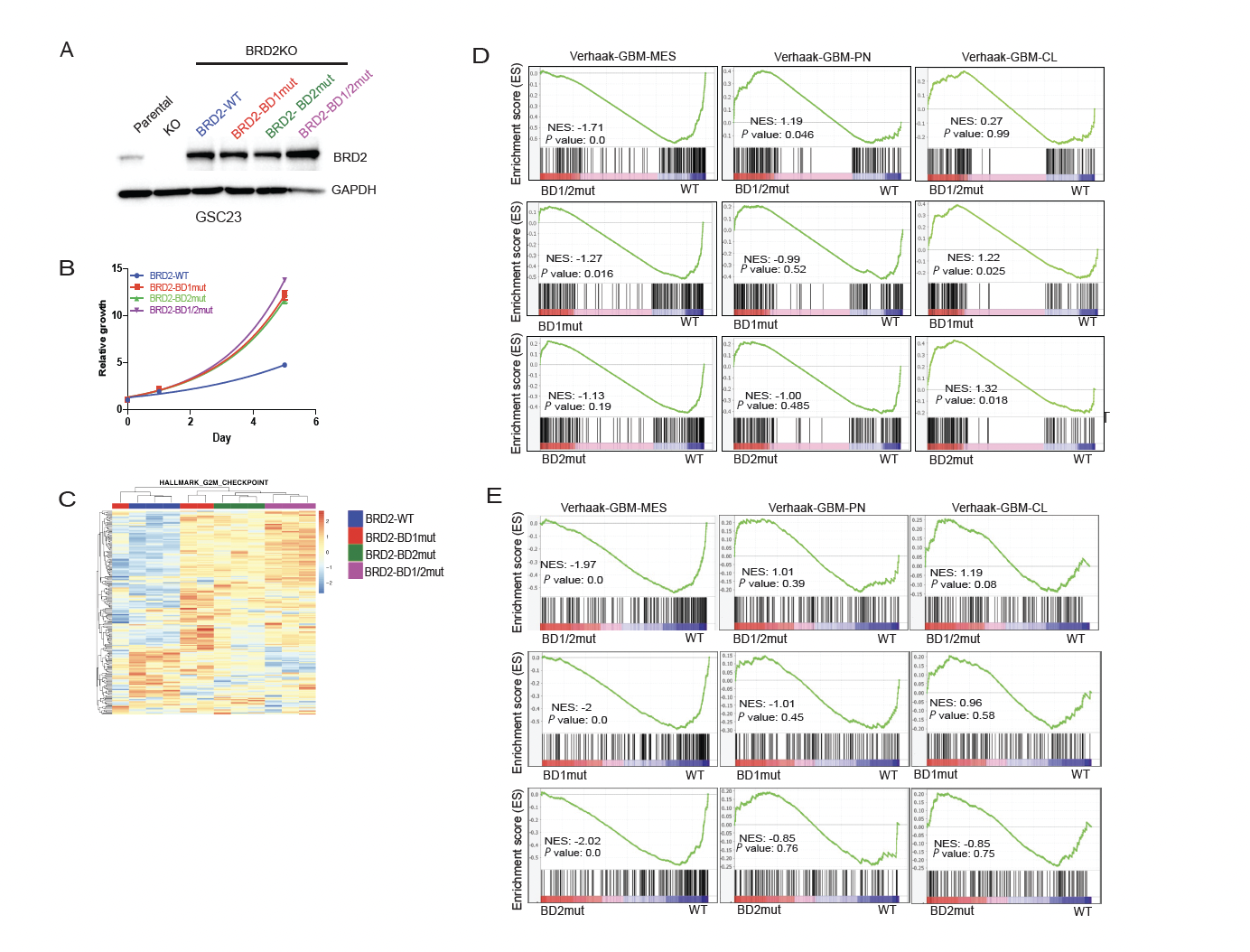


**Figure S4**. **A)** IB analysis of BRD2 deleted GSC23 cells rescued with BRD2-WT (wildtype), BRD2-BD1mut (Y113F), BRD2-BD2mut (Y386F) and BRD2-BD1/2mut (Y113F/Y386F).  **B)** ATPlite assay showing cell growth differences in GSC23 cells expressing BRD2-BD mutants (n=3 per group; mean ± SD). **C)** Heatmap showing the expression of G2M_checkpoint gene expression in GSC23 cells expressing BRD2-BD mutants compared with BRD2-WT. **D & E)** GSEA enrichment plots of GSC11 (**D)** and GSC23 (**E**) cells expressing WT-BRD2 or BRD2-BD mutants gene lists versus queried gene lists are shown.

**Supplemental Figure 5**

**
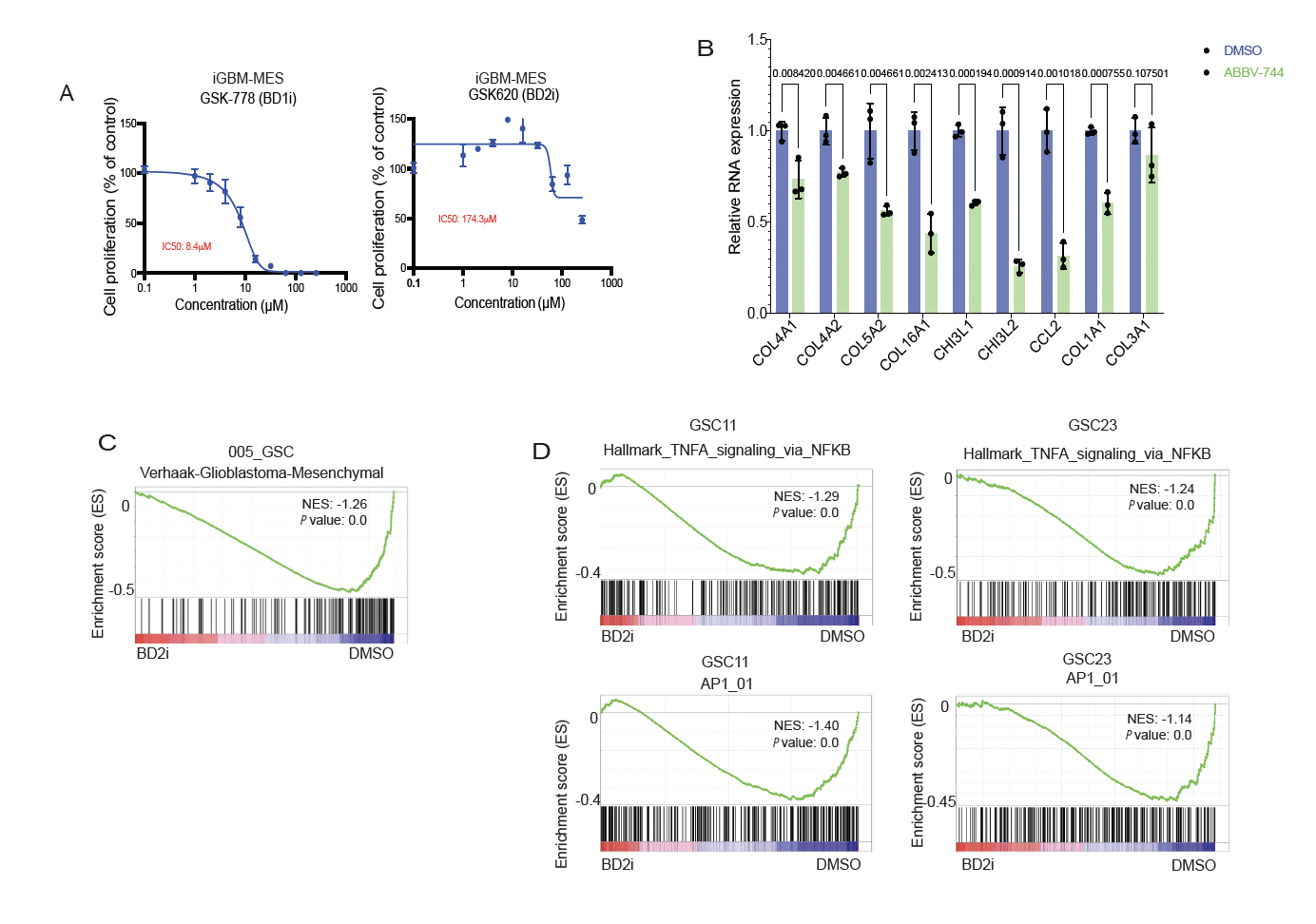
**

**Figure S5**. **A)** IC50 assays with ATPlite showing cell proliferation in GSCs treated with varying doses of BD1 inhibitor (GSK778) or BD2 inhibitor (GSK620) (n=3). **B)** qPCR analysis of MES gene expression in GSC11 GSCs treated with ABBV-744 (0.5μM, 24h). (n=3; mean ± SD). **C)** GSEA graphs from bulk RNAseq in 005GSCs cells treated with DMSO or GSK620 (5μM, 24h). **D)** GSEA graphs from bulk RNAseq in GSC11 and GSC23 cells treated with DMSO or GSK620 (0.5-5μM, 24-48h).

**Supplemental Figure 6**

**
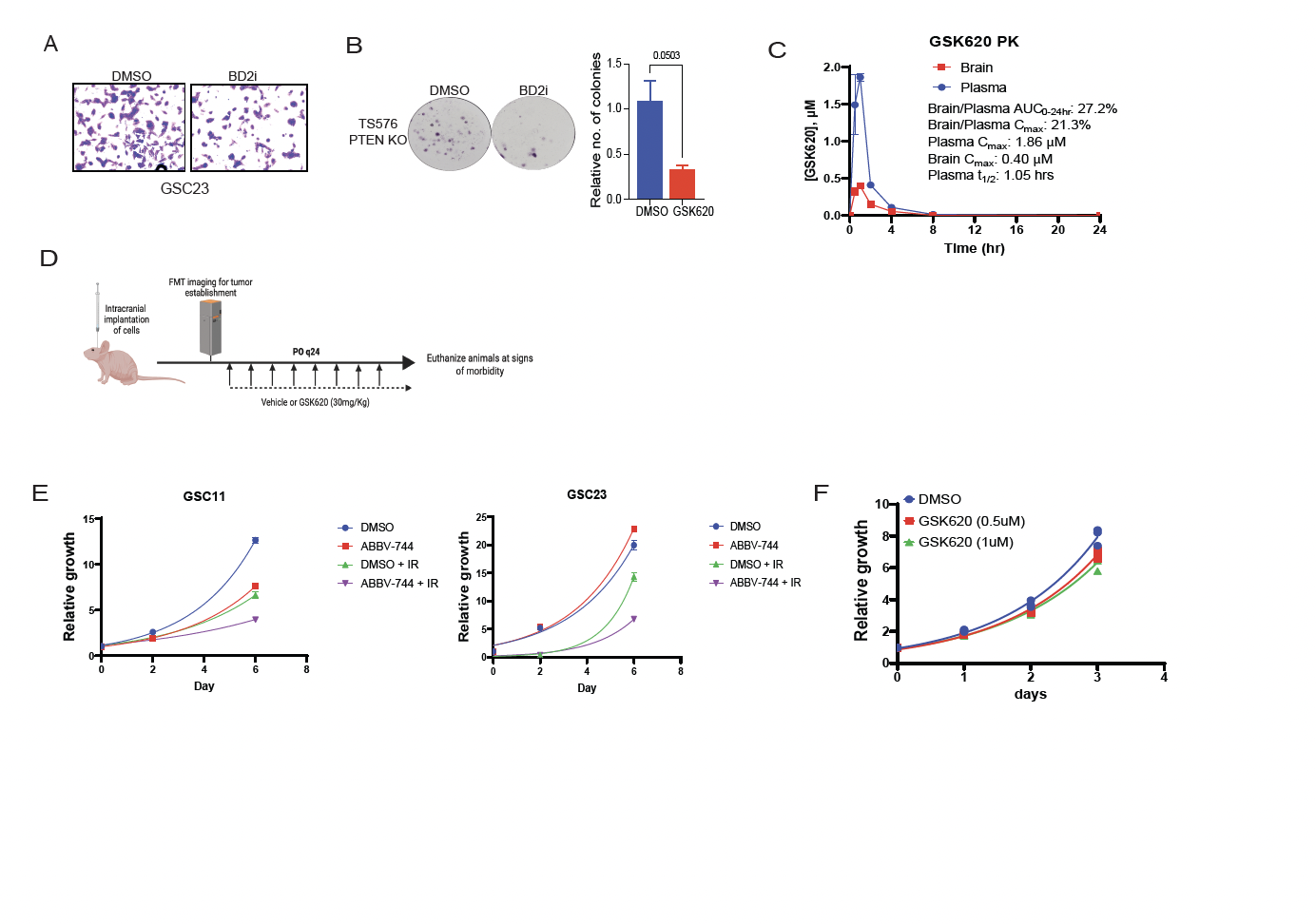
Figure S6**. **A)** Matrigel invasion assay comparing GSC23 cells treated with DMSO or GSK620 (2 μM, 24h). Invasive cells were quantified across five random fields and expressed as relative percentage (n = 3; mean ± SD). Scale bar, 100μm. **B)** Clonogenic assay with for TS576 PTEN KO cells treated with DMSO or GSK620 (2 μM, 24h). Data represents mean ± SD. **C)** PK analysis for GSK620 in mice. **D)** Experimental pre-clinical drug trial design: Mice were orthotopically transplanted with iRFP-720-labelled GSC11 GSCs (2 x 105 cells). Tumor burden was assessed by FMT imaging and mice were randomly assigned to vehicle or GSK620 treatment (30mg/kg). **E)** ATPlite assay of GSCs treated with DMSO or ABBV-744 (0.5μM, 24h) were exposed to one dose of IR (2-4Gy) and assessed for cell growth on indicated timepoints. (n=3 per group; mean ± SD). **F)** ATPlite of NPCs treated with varying doses of GSK620. (n=3 per group; mean ± SD).

**Supplementary Methods:**

**GSK620 detection**

Chromatographic separations were performed on a 100 x 2.1 mm Phenomenex Kinetex C18 column (Kinetex) using the 1290 Infinity LC system (Agilent). The mobile phase was composed of solvent A: 0.1% formic acid in Milli-Q water, and B: 0.1% formic acid in acetonitrile. Analytes were eluted with a gradient of 5-95% B (1-15 min), 95% B (15-20 min), and then returned to 5% B for 5 min to re-equilibrate between injections. Injections of 20 µL into the chromatographic system were used with a solvent flow rate of 0.10 mL/min.

Mass spectrometry was performed on the 6460 triple quadrupole LC/MS system (Agilent). Ionization was achieved by using electrospray in the positive mode and data acquisition was made in multiple reactions monitoring (MRM) mode. Two MRM transitions were used for GSK620: m/z 325→ 169 and 325→ 247 with fragmentor voltage of 85V, and collision energy of 17 and 5 eV, respectively. Analyte signal was normalized to the internal standard and concentrations were determined by comparison to the calibration curve (0.5, 5, 50, 250, 500, 2000 nM). GSK620 brain concentrations were adjusted by 1.4% of the mouse brain weight for the residual blood in the brain vasculature as described by Dai et al.^39^.

**Pharmacokinetic studies**

Whole blood from mice was centrifuged to isolate plasma. GSK620 was isolated by liquid-liquid extraction from plasma: 50 µL plasma was added to 2 µL internal standard and 3-fold volume acetonitrile. Mouse brain tissue was washed with 2 mL cold PBS and homogenized using a sonicator with fresh 2 mL cold PBS. GSK620 was then isolated and reconstituted in a similar manner by liquid-liquid extraction: 100 µL brain homogenate was added to 2 µL internal standard and 3-fold volume acetonitrile. After vortex mixing, the samples were centrifuged. The supernatant was removed and evaporated by a rotary evaporator and reconstituted in 100 µL 50:50:0.1 water:acetonitrile:formic acid**.**

**Generation of RELA K310R and PTEN KO GSCs**

pSpCas9(BB)-2A-GFP (px458) plasmid was obtained from Addgene (plasmid #48138). The designated sgRNA sequences for each of the targeted genes were cloned into px458 using combinations of top and bottom oligonucleotides listed below.

PTEN – guide 1-top: 5’ – CACCGGAATTTACGCTATACGGAC – 3’

PTEN – guide 1-bottom: 5’ – AAACGTCCGTATAGCGTAAATTCCC -3’

PTEN – guide 2-top: 5’ – CACCGAACAAGATCTGAAGCTCTAC – 3’

PTEN – guide 2-bottom: 5’ – AAACGTAGAGCTTCAGATCTTGTTC – 3’

RELA-K310R-top: 5’ – CACCGCTTCTTCATGATGCTCTTGA – 3’

RELA-K310R-bottom: 5’ – AAACTCAAGAGCATCATGAAGAAGC - 3’

Each pair of top and bottom oligonucleotides were phosphorylated and annealed by incubating 10 µM of each with 1 × T4 DNA ligase buffer (New England Biolabs), 5U T4 polynucleotide kinase (New England Biolabs) at 37 °C for 30 min, 95 °C for 5 min and by cooling down to 25 °C at 0.1 °C/s using a thermocycler. Annealed oligonucleotides were cloned into px458 by incubating 25 ng plasmid, 1 μM annealed oligonucleotides, 1× CutSmart buffer (New England Biolabs), 1 mM ATP (New England Biolabs), 10U BBSI-HF (New England Biolabs) and 200U T4 ligase (New England Biolabs) at 37 °C for 5 minutes, 23 °C for 5 min for 30 cycles. Correct cloning of each sgRNA sequence was confirmed by Sanger sequencing using U6 sequencing primer: 5′- GATACAAGGCTGTTAGAGAGATAATT-3′.

A single-stranded oligo DNA nucleotides (ssODNs) listed below was used to introduce the point mutation into the RELA gene.

RELA-K310R-ssODN

5’ -CCTTACTTTCCCAGACGATCGTCACCGGATTGAGGAGAAACGTAAAAGGACATATGAGACATTCCGCAGCATCATGAAGAAGAGTCCTTTCAGCGGTGAGATGGGGACTGGGAAAGCCAGAGAGGAA - 3’

GSCs were dissociated to single cells using Accutase (Innovative Cell Technologies). The dissociated GSCs (1 × 10^6^ cells) were resuspended in 100 μl of supplemented solution of the Human Stem Cell Nucleofector Kit 1 (Lonza) containing a combination of the px458 plasmid targeting each gene and the ssODN and then electroporated using B-016 program of Nucleofector 2b (Lonza). The electroporated GSCs were cultured for 48 h followed by Single cell sorting of GFP-positive cells (SH800, SONY) into 96-well plates. For screening duplicated 96-well plates were lysed using QuickExtract DNA Extraction Solution (Epicenter) and the following primers were used to confirm the edited GSCs.

RELA – forward: 5’ – GGACATATGAGACCTTCCGC – 3’

RELA – reverse: 5’ – AGGGCTAGGTCAGTTCTCAG – 3’

**Generation of BRD2 Knockout in GSCs**

To generate BRD2 knockout GSCs, we used CRISPR-Cas9 ribonucleoprotein (RNP) delivery via synthetic guide RNAs and recombinant SpCas9. sgRNAs targeting exon regions of the *BRD2* gene were purchased from Synthego (Multiplex sGRNA gene knockout kit). GSCs were dissociated to single cells using Accutase (Innovative Cell Technologies). The dissociated GSCs (1 × 10^6^ cells) were resuspended in 100 μl of supplemented solution of the Human Stem Cell Nucleofector Kit 1 (Lonza) containing a combination of sgRNAs and SpCas9 and then electroporated using B-016 program of Nucleofector 2b (Lonza). The electroporated GSCs were cultured for 48 h followed by Single cell sorting (SH800, SONY) into 96-well plates. For screening duplicated 96-well plates were lysed using QuickExtract DNA Extraction Solution (Epicenter) and knockout GSCs were confirmed with PCR followed by western blot. Following guides were used: sgRNA#1: GGUAACUCGUCCUGGCUUUU, sgRNA#2: AUCAGUUCGCAUGGCCAUUC, sgRNA#3: UGAGAGCCCCACAAUGGCUU

**Western blotting**

Extracts were separated using gel electrophoresis and transferred via wet transfer onto a PVDF membrane. The membrane was blocked with 5% milk in TBST and probed with primary antibodies in 5% BSA at 1:1,000 dilution overnight at 4°C and secondary HRP antibodies in 5% milk at 1:10,000 for 1 hour at RT. Signal was assessed via chemiluminescence with SuperSignal West Pico PLUS substrate (Thermo Fisher, #34580) and visualized on a ChemiDoc MP system (Bio-Rad).

I**HC**

Formalin-fixed, paraffin-embedded (FFPE) tissue was prepared by the Histology Core Facility at UCSD pathology. Five μm thick tissue sections were immunostained for Human Nucleoli (clone NM95; Abcam; ab190710) and Iba-1 (Wako; 019-19741). Slides were immuno­stained using a Ventana Discovery Ultra (Ventana Medical Systems,). Antigen retrieval was performed using CC1 (tris-based; pH 8.5) for 64 mins at 95°C. The primary antibody NM95 was incubated with the slides for 32 min at 37°C. The secondary antibody, OmniMap anti-Rb-HRP (#760-4311, Ventana), was incubated on the sections for 12 min at 37°C. NM95 presence was visualized using Ventana purple chromagen for 32 minutes. The slides were treated with Ventana CC2 (pH 6.5) for 24 min at 95^o^C to remove both primary and secondary antibodies. Subsequently the slides were incubated with the second primary against Iba-1 for 32 min at 37^o^C followed by Ultra-Map anti-Rb-HRP for 12 min at 37^o^C and incubated with the green chromagen (Ventana) for 20 min. IHC staining was followed by hematoxylin as a counterstain. Slides were rinsed, dehydrated through alcohol and xylene and cover slipped. Representative images from each immunostained section were taken with a Keyence BZ-X700 microscope and analyzed with BZ-X Analyzer Keyence software.
